# Molecular detection of *Coxiella Burnetii* in ovine abortions: evidence from a three-year surveillance in an endemic region

**DOI:** 10.1016/j.vas.2025.100550

**Published:** 2025-12-04

**Authors:** Pegah Sagha Nosrati, Khadijeh Hashemi, Narges Khaleghnia, Mehrdad Mohri, Pezhman Mirshokraei

**Affiliations:** a Department of Clinical Sciences, School of Veterinary Medicine, Ferdowsi University of Mashhad, Mashhad, Iran; b Stem Cell Biology and Regenerative Medicine Research Group, Research Institute of Biotechnology, Ferdowsi University of Mashhad, Mashhad, Iran; c Centre of Excellence in Ruminant Abortion and Neonatal Mortality, School of Veterinary Medicine, Ferdowsi University of Mashhad, Mashhad, Iran

**Keywords:** Abortion, *Coxiella burnetii*, Prevalence, real-time PCR, Sheep

## Abstract

*Coxiella burnetii*, an obligate intracellular coccobacillus, is a major cause of abortion in livestock and a re-emerging zoonotic pathogen responsible for Q fever in humans. Iran, particularly Khorasan-e-Razavi Province, is considered endemic for Q fever. Rapid and accurate detection of *C. burnetii* in animal shedders, especially asymptomatic ones, is essential to control its spread among animals and from animals to humans.

This study provides the first multi-year (2020–2022) quantitative assessment of *C. burnetii* infection load in ovine abortions in this endemic region. The objectives were to: (i) develop and validate a region-specific real-time PCR assay targeting the IS1111a gene through sequencing of the amplified fragment; (ii) quantify pathogen load using a standard curve derived from a sequenced reference fragment; and (iii) evaluate associations between prevalence and factors such as year, season, geographic location, and confirmed co-infections with other abortifacient agents. Liver samples from 116 aborted sheep fetuses were examined by real-time PCR, revealing a *C. burnetii* prevalence of 53.4%, while in 54.8% of these positive cases, *C. burnetii* was identified as the sole infectious pathogen contributing to abortion. A significant association (p < 0.05) between sampling year and prevalence was observed, with a rising trend over time. The high prevalence of *C. burnetii* and its role in abortion cases underscore the need for enhanced surveillance and control programs to mitigate the spread and impact of this pathogen in endemic regions.

## 1 Introduction

*Coxiella burnetii* is a small Gram-negative coccobacillus and an obligate intracellular bacterium, the causative agent of Q fever in humans and animals (Günaydin et al., 2015). Based on 16S rRNA sequence analysis, *C. burnetii* belongs to the Gamma subdivision of Proteobacteria within the order Legionellales and family Coxiellaceae. The pathogen can resist difficult physical and chemical environmental stresses, and infects a broad range of hosts, including ruminants, pets, birds, and arthropods, with cattle, sheep, and goats being the main reservoirs (Eldin et al., 2017; Maurin & Raoult, 1999). In livestock, it frequently causes reproductive disorders such as abortion, retained fetal membranes, endometritis, infertility, and low birth weight, which result in major economic losses (Bouvery et al., 2003; Rodolakis, 2009).

Ruminants shed high loads of *C. burnetii* in birth products, milk, feces, vaginal mucus, and urine (Eldin et al., 2017; Rodolakis, 2009; Guatteo et al., 2011). Transmission to humans typically occurs through inhalation of contaminated aerosols or consumption of unpasteurized dairy products (Eldin et al., 2017; Angelakis & Raoult, 2010; Parker et al., 2006). The disease is often underreported in humans, but increasing case numbers worldwide indicate its re-emergence (Arricau-Bouvery et al., 2006). Occupational risk is high among farmers, veterinarians, slaughterhouse workers, and laboratory staff, particularly during parturition or handling of infected tissues (Million & Raoult, 2015).

Laboratory diagnosis of Q fever has evolved significantly. Older techniques such as serology, histopathology, and smears (e.g., Stamp’s modified Ziehl-Neelsen staining) are still in use but suffer from low sensitivity and specificity (Kılıç et al., 2016; Vaidya et al., 2010). Several studies have shown that smear staining or immunohistochemistry often fail to detect cases later confirmed by PCR (Hazlett et al., 2013; Reber et al., 2012). In contrast, probe-based real-time PCR assays (e.g., TaqMan systems) have become the gold standard for detecting *C. burnetii*, offering superior sensitivity and quantitative capabilities (Jones et al., 2010; Roest et al., n.d.). For instance, Jones et al. (2010) demonstrated that IS1111-targeting TaqMan qPCR could detect as few as ∼10 genomic copies per reaction (Jones et al., 2010). Hazlett et al. (2013) found that 69–75 % of sheep and goat abortions were PCR-positive, while only a minority showed histological lesions or were confirmed by traditional pathology (Hazlett et al., 2013).

Despite several serological and molecular surveys, quantitative data describing the infection load, strain validation, and multi-factorial epidemiological patterns of *C. burnetii* in livestock abortions in Iran remain scarce. In endemic provinces such as Khorasan Razavi, limited diagnostic capacity and lack of region-specific molecular validation restrict effective surveillance and control programs. Therefore, this study was designed with four complementary objectives: (1) to establish and validate a SYBR-based quantitative PCR assay targeting the multi-copy *IS1111a* gene and to confirm the amplified fragment by Sanger sequencing for regional strain specificity; (2) to quantify *C. burnetii* copy numbers in fetal liver tissues collected over three years (2020–2022); and (3) to evaluate associations between *C. burnetii* prevalence and potential risk factors including season, year, geographic region, and confirmed co-infection with other abortion-inducing pathogens. This investigation was conducted at the Center of Excellence in Ruminant Abortion and Neonatal Mortality (COE-RAM), Ferdowsi University of Mashhad, Iran.

## 2 Materials and methods

### 2.1 Sample collection and preparation

**Fig. 1:**
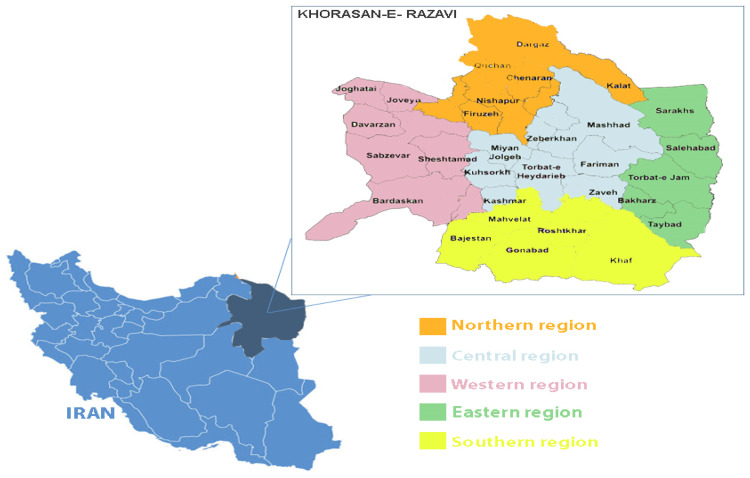
Map of Khorasan Razavi province showing sampling locations divided by regions *Sampling sites within each region correspond to the local administrative divisions (counties) where animal abortion cases were investigated for *Coxiella burnetii* infection. The small map of Iran indicates the location of Khorasan-e Razavi province (dark blue).

A total of 116 DNA samples extracted from the liver tissue of aborted ovine fetuses were obtained from the biobank of the *Center of Excellence in Ruminant Abortion and Neonatal Mortality (COE-RAM)*, Ferdowsi University of Mashhad, Iran. These archival samples were collected over three years (2020–2022) from different regions of Khorasan Razavi Province (Northern, Central, Eastern, and Western) as illustrated in Fig. 1.

Local veterinarians or farmers had submitted each fetus following field abortion events. For molecular analysis, liver tissue was selected as the primary matrix for both conventional and real-time PCR assays. Each sample was labeled with metadata including the year, season (spring, summer, autumn, or winter), and geographic origin. Additional diagnostic testing for other abortion-causing agents was performed on the same extracted DNA and corresponding samples according to the standard molecular and bacteriological protocols of the COE-RAM diagnostic center. These included bacteriological culture for Brucella spp., Campylobacter spp., and *Escherichia coli*, and molecular detection (PCR) for *Chlamydia abortus* and *Toxoplasma gondii*. DNA was extracted from liver tissue using the AddPrep Genomic DNA Extraction Kit (Addbio® Inc., Korea), according to the manufacturer’s instructions, and was stored at −20°C until further use.

Ethical approval (IR. UM. REC. 1401.065) for this study was issued in accordance with the ethical guidelines for research at the School of Veterinary Medicine, Ferdowsi University of Mashhad.

### 2.2 Designing primers

To detect *C. burnetii* species, a pair of primers targeting the IS1111a transposase gene was designed using Beacon Designer (version 8.10, Premier Biosoft, USA). In silico specificity was examined using the Basic Local Alignment Search Tool (BLAST) in the GenBank database and SnapGene software (version 3.2.1, USA). Table 1 presents the characteristics of the primers applied in the present study.

**Table 1:** Characteristics of Designed Forward and Reverse Primers Targeting the *IS1111a* gene.

Primer	Band size	Accession number
Fw: 5′-CGTGCTCAGTATGTATCC-3′	98 bp	*IS1111a* M80806
Rv:5′-CCCATAAACGTCCGATAC-3′		

### 2.3 DNA amplification

To prepare molecular standards, a conventional PCR test using designed primers was performed on DNA extracted from a confirmed positive liver sample obtained from the Center of Excellence in Ruminant Abortion and Neonatal Mortality (COE-RANM), Ferdowsi University of Mashhad. This initial PCR was not for diagnostic purposes, but to amplify the desired 98 bp product. PCR reactions were carried out in 25 μL mixtures containing 10 µL of Taq 2x Master Mix Red (Ampliqon A/S, Denmark), 5 µL of template DNA, one μL of each primer at 10 μM concentration (Metabion International AG, Planegg, Germany), and three μL of UltraPure™ DNase/RNase-Free Distilled Water. The thermal profile included: 94°C for 15 min; 40 cycles at 94°C for 30 sec, 59°C for 30 sec, and 72°C for 30 sec; with a final extension at 72°C for 10 min.

The resulting 98 bp PCR product was visualized by electrophoresis on a 2 % agarose gel. The target band was excised, gel-purified using a commercial extraction kit (DENA ZIST ASIA®, Iran, Mashhad), and quantified using a NanoDrop™ 2000/2000c spectrophotometer (Thermo Fisher Scientific, Germany). The same purified DNA was also submitted for Sanger sequencing to confirm specificity. The amplified sequences were aligned using SnapGene software against the NCBI GenBank database to confirm the identity of *Coxiella burnetii*. This purified and sequence-verified DNA served as the molecular standard.

### 2.4 Real-time PCR and standard curve generation

MicPCR software (mic-PCR®, Applied Biomolecular Systems Co., Australia) was used to generate a standard curve for quantification. The DNA used for this standard curve was a purified, sequence-verified amplicon from the previous step, and the DNA copy number was calculated using the Integrated DNA Technologies (IDT) online calculator based on its molecular weight.

Ten-fold serial dilutions of the quantified and sequence-confirmed DNA were prepared, and each dilution was tested in two replicates. The Ct values and corresponding DNA concentrations were used by the software to generate the standard curve, which was subsequently used to calculate copy numbers in the test samples.

Real-time PCR assays were carried out in 12 μL reaction mixtures containing five μL of Real Q Plus 2x Master Mix Green without ROX (Ampliqon A/S, Odense, Denmark), 0.5 μL each of forward and reverse primers at 10 μM concentration (Metabion International AG, Planegg, Germany), four μL of extracted sample DNA, and two μL of UltraPure™ DNase/RNase-Free distilled water. The thermal cycling conditions are detailed in Table 2.

**Table 2:** The thermal cycling program used for amplification of the transposase gene of *IS1111a*.

Activation: 95°C for 15 min			
40 cycles	Denaturation	95°C	30 s
	Annealing	58°C	30 s
	Extension	72°C	30 s
Melting: From 60°C to 95°C at 0.3°C /second			

The auto baseline and threshold functions in the mic-PCR® Software v2.6.4 were used to determine Ct values. All samples were tested in two replicates. A sample was considered positive if the Ct value was ≤35 and the melting temperature fell within 83.4–84.4°C. PCR results are expressed as copy numbers per microliter (copies/µL).

### 2.5 Data analysis

Statistical analysis was performed using two complementary approaches. For categorical comparisons, the chi-square test was applied to assess associations between *Coxiella burnetii* detection and four variables: (1) year of sampling, (2) season of sampling, (3) geographic location within Khorasan Razavi province (North, East, West, Center), and (4) co-infection with other abortifacient agents. Depending on sample size, either the standard chi-square test or Fisher’s exact test was used where appropriate. A significance level of p < 0.05 was considered indicative of statistical significance.

In addition, a multinomial logistic regression model was employed to investigate associations between the independent variables (year, season, and region) and abortion etiology, the dependent variable categorized as 0 = no Coxiella infection, 1 = Coxiella alone, and 2 = co-infection with Coxiella and other agents. The analysis was performed in Python using the statsmodels library (version 3.12.3) on a dataset of 116 observations. Statistical significance was established at p < 0.05.

## 3 Result

### 3.1 Molecular results

By evaluating the weight of the produced fragment on a 2 % agarose gel, the isolate was identified by 98 bp amplicons as *Coxiella Brunetii*. Results of Sanger sequencing indicated that the positive sample was *Coxiella Brunetti.*

### 3.2 Standard curve analysis

**Fig. 2:**
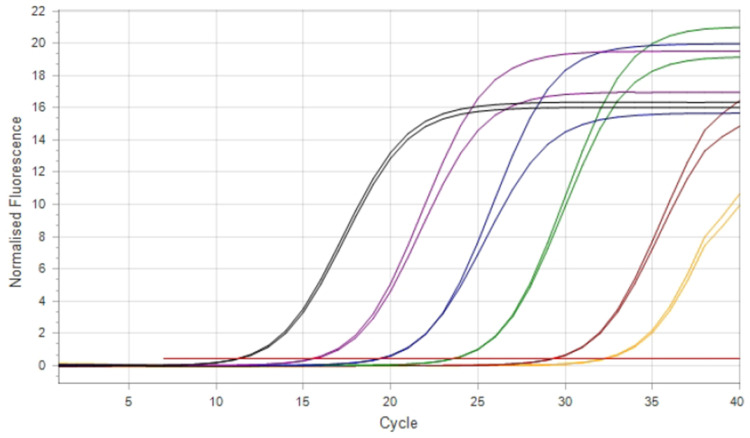
Amplification plot of serial dilutions used for standard curve generation**.** Note: Color codes represent template concentrations as follows: Black (10⁻³): 6.43 × 10⁷ copies/µL; Purple (10⁻⁴): 6.43 × 10⁶ copies/µL; Dark blue (10⁻⁵): 6.43 × 10⁵ copies/µL; Green (10⁻⁶): 6.43 × 10⁴ copies/µL; Maroon (10⁻⁷·⁵): 9.6 × 10² copies/µL; Yellow (10⁻⁹): 6.4 × 10¹ copies/µL; Red: Non-template controls.

**Fig. 3:**
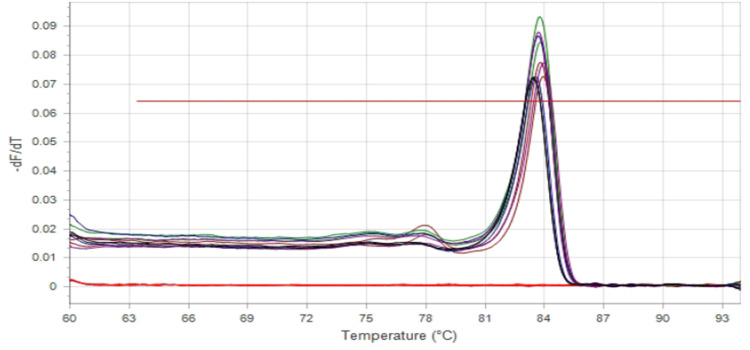
Melting curve analysis of SYBR Green real-time PCR products for the *IS1111* gene.

A standard curve was generated using ten serial dilutions of a *Coxiella burnetii* molecular standard containing 6.4 × 10¹⁰ copies/µl of the *IS1111a* gene. SYBR Green-based real-time PCR was used to assess assay performance under the study’s conditions. Each dilution was tested in duplicate, and intermediate points (e.g., 10⁻⁷^.^⁵) were included to improve linearity and curve fitting. No amplification was observed in non-template controls, confirming the absence of contamination (Figs. 2, 3).

Melting curve analysis confirmed the specificity of the SYBR Green PCR assay. A single, sharp peak was observed at approximately 83.8°C, corresponding to the melting temperature (Tm) of the *IS1111* amplicon, indicating the absence of primer-dimers or nonspecific amplification. Non-template controls (red) showed no detectable peaks, validating the assay specificity and purity of the amplification products.

**Fig. 4:**
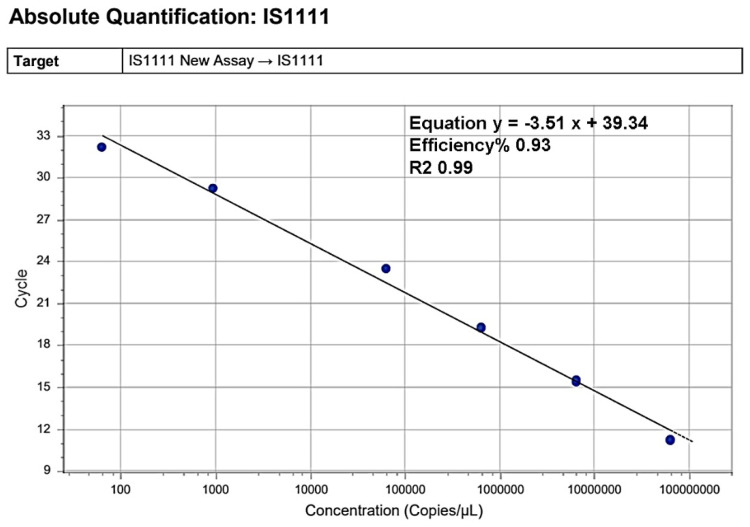
Standard curve for absolute quantification of the *IS1111* gene.

The standard curve was constructed by plotting quantification cycle (Cq) values against the logarithm of *C. burnetii IS1111* gene copy numbers, using serial tenfold dilutions ranging from 6.43 × 10⁷ to 6.4 × 10¹ copies/µL. A strong linear relationship (R² = 0.99) confirmed the assay's high efficiency and precision. The resulting equation, y = –3.51x + 39.34, corresponded to an amplification efficiency of 93 %, and was used for absolute quantification of unknown samples (Fig. 4).

### 3.3 Statistical analysis

Out of 116 aborted sheep fetuses tested, 62 (53.4 %) were positive for *Coxiella burnetii* by real-time PCR. The annual positivity rates were 25 % in 2020, 65.8 % in 2021, and 85.1 % in 2022, indicating a marked upward trend over time (Table 4). Among positive cases, 45.2 % (n = 28) were co-infected with other major abortifacient agents, including Brucella, Campylobacter, *Chlamydia abortus, T. gondii*, or *E. coli* (Table 5).

**Table 4:** Distribution of abortion cases by season, location, and year based on *C. burnetii* detection and co-infection status.

		Abortion with *C. burnetii* positive result (n=62)		Abortion with *C. burnetii* negative result (n= 54)	Total tested sample (n=116)
		Only *C. burnetii* positive (n=34)	*C. burnetii* with Co-infection of other abortifacient agents (n= 28)		
Season	spring	2	2	3	7
	summer	4	1	4	9
	fall	17	16	19	52
	winter	11	9	28	48
Location	North	20	11	33	64
	West	4	7	8	19
	East	4	2	7	13
	Central	6	8	6	20
Year	2020	4	8	36	48
	2021	17	10	14	41
	2022	13	10	4	27

**Table 5:** Frequency of infectious agents detected in aborted sheep fetuses (2020–2022).

No. of Coxiella-positive samples	Infectious profile	No. of samples	Frequency ( %)*
Only *Coxiella burnetii*	—	34	54.84
Co-infection with *Coxiella burnetii*	*Brucella spp.*	12	19.35
	*Campylobacter spp.*	6	9.68
	*Toxoplasma gondii*	1	1.61
	*Chlamydia abortus*	3	4.84
	*E. coli*	3	4.84
	*Brucella spp. + Chlamydia abortus*	2	3.23
	*Campylobacter spp. + Toxoplasma gondii*	1	1.61

⁎ Percentages were calculated relative to the total number of *Coxiella*-positive cases (n = 62).

The results of this study were examined in four aspects using the Chi-square test. The results indicated a significant relationship (p<0.05) between the three consecutive years of sampling and the disease prevalence. Also, in pairwise comparisons between the years, there was a significant relationship between prevalence in the years 2020 and 2021 with Risk Difference (RD): 40.85 % (CI: 21.86 % - 59.85 %) and Odds Ratio (OR): 5.79 (CI: 2.31 - 14.50), and between 2020 and 2022 with RD: 60.19 % (CI: 42.03 % - 78.34 %) and OR: 17.25 (CI: 4.96 - 60.01), whereas no significant difference was observed between 2021 and 2022.

The Chi-square test results for the association between the prevalence of *C. burnetii* and the independent variables (Season, Geographical location, and Co-infection) were not statistically significant. To further evaluate predictors of abortion etiology, a multinomial logistic regression was performed. The dependent variable was categorized as: 0 = no *C. burnetii*, 1 = *C. burnetii* infection only, and 2 = *C. burnetii* + co-infection. Independent variables included year, season, and location. The model was statistically significant overall (χ² = 46.49, df = 16, p = 8.1 × 10⁻⁵) with a pseudo-R² of 0.189. Among all predictors, only year had a significant effect. Compared to 2020, the odds of *C. burnetii*-only infection increased significantly in 2021 (OR = 18.75; 95 % CI: 4.34–81.00) and 2022 (OR = 29.67; 95 % CI: 5.43–162.00). Similarly, co-infection odds were elevated in 2021 (OR = 4.16; p = 0.037) and 2022 (OR = 10.16; p = 0.004). Neither season nor location showed statistically significant associations with abortion etiology in any reference configuration.

## 4 Discussion

*Coxiella burnetii* has been recognized as a significant cause of animal abortion and a zoonotic agent with potential for human transmission. Q fever outbreaks linked to infected sheep have been reported in several countries, including Bulgaria, Croatia, France, Germany, Italy, and Switzerland (Van den Brom et al., 2015; Esmaeili et al., 2019, Roest et al., 2011). Despite its known presence for over 50 years in Iran, the disease remains underdiagnosed due to the absence of a formal registration and reporting system and inadequate diagnostic or preventive protocols (Mohabbati Mobarez et al., 2017; Mobarez et al., 2020). This study aimed to determine the prevalence of *C. burnetii* in sheep aborted fetuses in Iran and represents the first use of quantitative PCR to evaluate infectious load.

Past Iranian studies have reported a prevalence of 17–20 % for *C. burnetii* in sheep herds (Dehkordi & Rafsanjani, 2012; Lorestani et al., 2015; Roshan et al., 2018). Esmaeili et al. (2022) found *C. burnetii* as the leading agent (22.7 %) among pathogens in aborted fetuses, followed by *Chlamydia abortus* and *Brucella melitensis*. A systematic review (2008–2016) across ten provinces estimated the prevalence in sheep milk at 3.79 %, with the highest in Khorasan Razavi (34.4 %) (Esmaeili et al., 2019). Another study in 2017 found 18.6 % positivity in milk samples (Mobarez et al., 2021). Other investigations reported 17.3 % positivity in aborted fetuses from COE-RAM (Abiri et al., 2016) and 21.3 % using IS1111a PCR, with Tehran showing the highest rate at 54.4 % (Mobarez et al., 2020).

Our study revealed 53 % positivity in 116 fetal liver samples—twice the prevalence found in previous Iranian studies. Factors contributing to this higher rate could include differences in sample types, sample size, timing, and primer design. In this study, the fetal liver was selected as the diagnostic tissue. While some studies utilize placenta or vaginal swabs, liver tissue has demonstrated comparable or even superior detection rates in small ruminants (Arricau-Bouvery et al., 2006; Berri et al., 2007; Gwida et al., 2014). This is likely due to the hematogenous route of *C. burnetii* transmission in utero, with the liver being among the first and most consistently infected organs (Klee et al., 2006; Guatteo et al., 2007).

A SYBR Green–based real-time PCR was selected over TaqMan due to its sensitivity, cost-effectiveness, and suitability for regional labs lacking probe-based systems. The IS1111a gene, with 7 to 110 copies per genome, was targeted to enhance PCR sensitivity (Klee et al., 2006; Sahu et al., 2020), and a local field sample was sequenced as a reference control positive, making the test more specific and reliable. Furthermore, this higher result could reflect increased pathogen circulation. Statistical analysis showed a notable and statistically significant increase in positivity from 2020 to 2022, suggesting an ongoing and expanding spread among sheep herds. Such trends may reflect unregulated animal movement, the persistence of the organism in contaminated environments, and gaps in routine surveillance. Given that *C. burnetii* can remain viable for months in dust and aerosols, even low levels of shedding can lead to recurrent environmental contamination and reinfection within herds (Álvarez-Alonso et al., 2020). This rise may indicate an increased risk of abortion in herds and zoonotic transmission to humans, posing a public health concern. Outbreaks have been reported across Europe, including the Netherlands, Bulgaria, France, Germany, Italy, and Switzerland (Álvarez-Alonso et al., 2018; Esmaeili et al., 2019; Van den Brom et al., 2015). Infected animals shed enormous quantities of *C. burnetii* in placental tissues, amniotic fluid, milk, urine, and feces, and viable bacteria can be detected in barn dust long after abortion events (Berri et al., 2007; Joulié et al., 2015).

An additional aim of the study was to evaluate the pathogenic threshold of *C. burnetii*. Detection of the organism alone does not confirm disease causation, and PCR positivity may not reflect actual pathogenicity (Hazlett et al., 2013; Berri et al., 2001; Berri et al., 2007). The current best practice is to use IS1111 qPCR for screening, but interpret results in light of Ct value and sample type. A very low bacterial load (high Ct) should be interpreted with caution: it may represent environmental contamination rather than true infection of the fetus or placenta (Van den Brom et al., 2025). Hazlett et al. (2013) proposed a threshold of 3.78 × 10³ copies/μl to distinguish pathogenic from incidental detection, showing only 10 % of PCR-positive sheep met this clinical relevance threshold. In our study, 12 of 62 positive samples (in 2020–2022) exceeded this cut-off, showed abortion symptoms, and had no co-infection—supporting their likely pathogenic role. Multiple epidemiological factors can influence the prevalence of *C. burnetii*. These include herd size, location, management practices, animal age and breed, and co-infection with other abortifacient agents (Rad et al., 2014; Ramo et al., 2022; Selim et al., 2018; Keshavamurthy et al., 2020; Schimmer et al., 2011; Capuano et al., 2004). In accordance with seasonal effects, we observed the highest detection rates during winter, consistent with the lambing season when parturition events increase the chance of bacterial shedding and environmental spread (Schimmer et al., 2014; Yadav et al., 2021). In contrast, no statistically significant regional variation was detected. However, some studies found a geographic effect on prevalence (Rad et al., 2014; Schimmer et al., 2011); we, like Roshan et al. (2018), did not observe significant regional variation in our dataset.

Regarding co-infections, previous studies from Portugal and elsewhere found co-presence of *C. burnetii* with agents like *Chlamydia* in 16–24 % of abortion cases (Santos et al., 2022; Ramo et al., 2022). Similarly, in our study, 25 % of positive samples were co-infected. Although this was not statistically significant, it highlights the diagnostic challenge when multiple pathogens are involved. Importantly, the lack of association between *C. burnetii* and co-infection in our analysis does not rule out pathogenic interactions; it merely reflects the constraints of our sample and analysis. Further studies are warranted to explore these dynamics in depth.

This study has several limitations that should be acknowledged, including the exclusive use of SYBR Green qPCR without comparison to TaqMan assays, analysis of only fetal liver tissues due to sample constraints, and the absence of histopathology, which restricts confirmation of causality in co-infections.

## 5 Conclusion

The result of the present study highlights the increasing prevalence of *C. burnetii* in sheep herds in Khorasan Razavi, Iran. Furthermore, regarding the importance of screening *C. burnetii* as a causative agent of abortion in livestock, the qPCR assay proposed in this study provides a valuable tool for considering the cut point of the bacteria's pathogenicity, along with the insights for making risk assessment and the implementation of counteractive measures.

## Ehics

The project was found to be in accordance to the ethical principles and the national norms and standards for concluding Medical Research in Iran. Notice: 1. Although the proposal has been approved by the Biomedical Research Ethics Committee, meeting the professional and legal requirements is the sole responsibility of the Pl and other project collaborators. 2. This certificate is reliant on the proposal/documents received by this committee on 2021-10-30. The committee must be notified by the Pl as soon as the proposal/documents are modified.

## CRediT authorship contribution statement

**Pegah Sagha Nosrati:** Writing – original draft, Investigation, Data curation. **Khadijeh Hashemi:** Supervision, Methodology, Investigation. **Narges Khaleghnia:** Writing – review & editing, Writing – original draft, Resources, Methodology, Investigation. **Mehrdad Mohri:** Validation, Funding acquisition, Formal analysis. **Pezhman Mirshokraei:** Writing – review & editing, Validation, Supervision, Resources, Funding acquisition.

## Declaration of competing interest

The authors declare that they have no known competing financial interests or personal relationships that could have appeared to influence the work reported in this paper.
